# Supplementary material for: Simple and versatile imaging of genomic loci in live mammalian cells and early pre-implantation embryos using CAS-LiveFISH
Source: Sci Rep. 2021 Jun 9;11:12220. doi: 10.1038/s41598-021-91787-y (PMC8190065; doi:10.1038/s41598-021-91787-y)
Supplement: Supplementary file 1 — Supplementary Information 1. [file 41598_2021_91787_MOESM1_ESM.pdf]

Simple and versatile imaging of genomic loci in live mammalian cells and early pre-implantation embryos using CAS-LiveFISH

Yongtao Geng[1] and Alexandros Pertsinidis[1,*]

[1] Structural Biology Program, Memorial Sloan Kettering Cancer Center, New York, NY 10065, USA

* To whom correspondence should be addressed. Email: PertsinA@mskcc.org

Supplementary Data

1. **Supplementary Figures 1-10**
2. **Supplementary Material**

    DNA Sequences

3. **Supplementary Tables 1-3**

Supplementary Figures

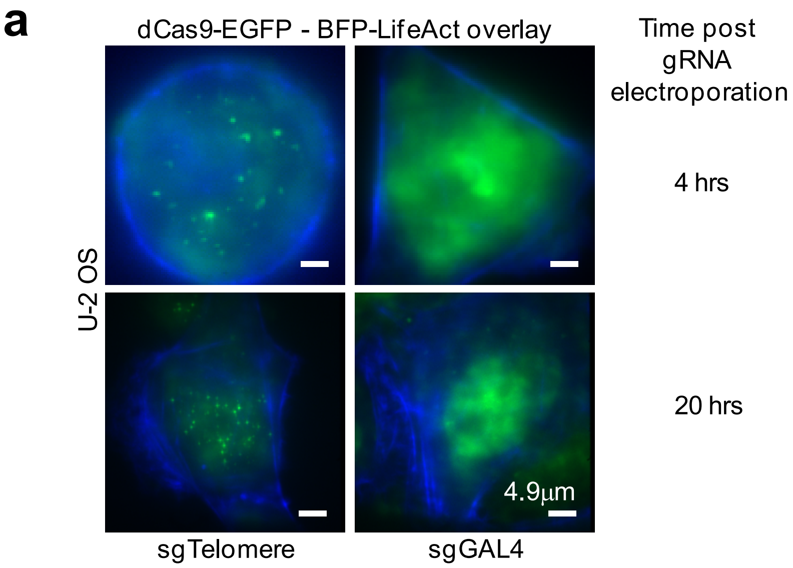

Supplementary Figure 1. Imaging of genomic loci by delivery of *in vitro* transcribed gRNAs. (**a**) Visualization of telomeres in U-2 OS cells stably expressing dCas9-EGFP, by electroporation. Examples are shown at 4 hrs and 20 hrs after electroporation. No nuclear puncta are observed for an sgGAL4 non-targeting control.

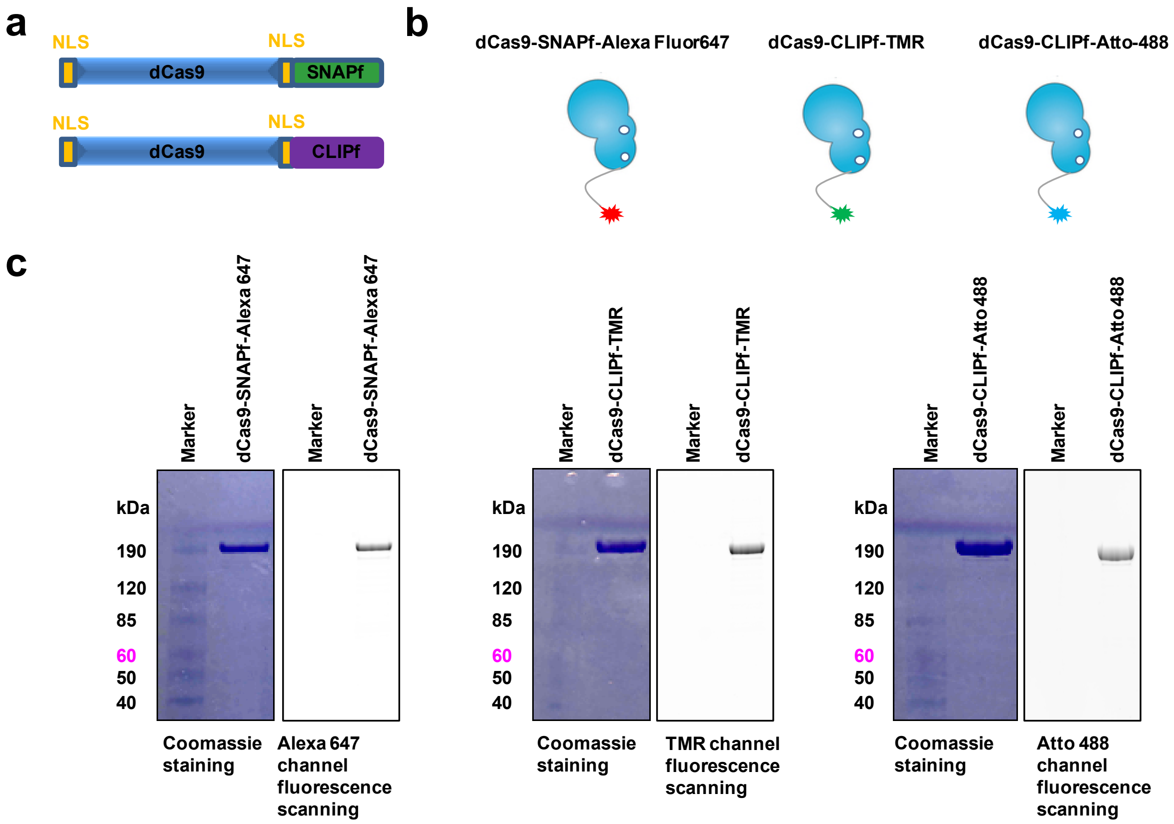

Supplementary Figure 2. Fluorescent dCas9 proteins. (**a, b**) Schematic of dCas9-SNAPf and dCas9-CLIPf constructs. (**C**) SDS-PAGE showing purified and fluorescently labelled dCas9 proteins.

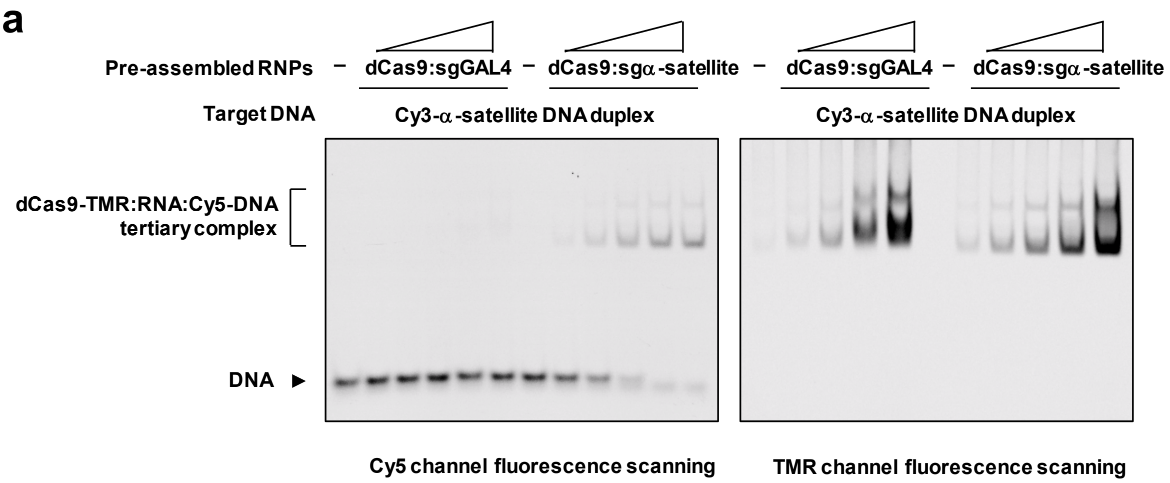

Supplementary Figure 3. Electrophoretic mobility shift assays. (**a**) Pre-assembled dCas9-TMR RNPs were incubated with Cy5-DNA duplexes and ternary complexes are resolved from free DNA using native SDS-PAGE. Binding is observed for the targeting gRNA (sgα-satellite) but not for the non-targeting control (sgGAL4).

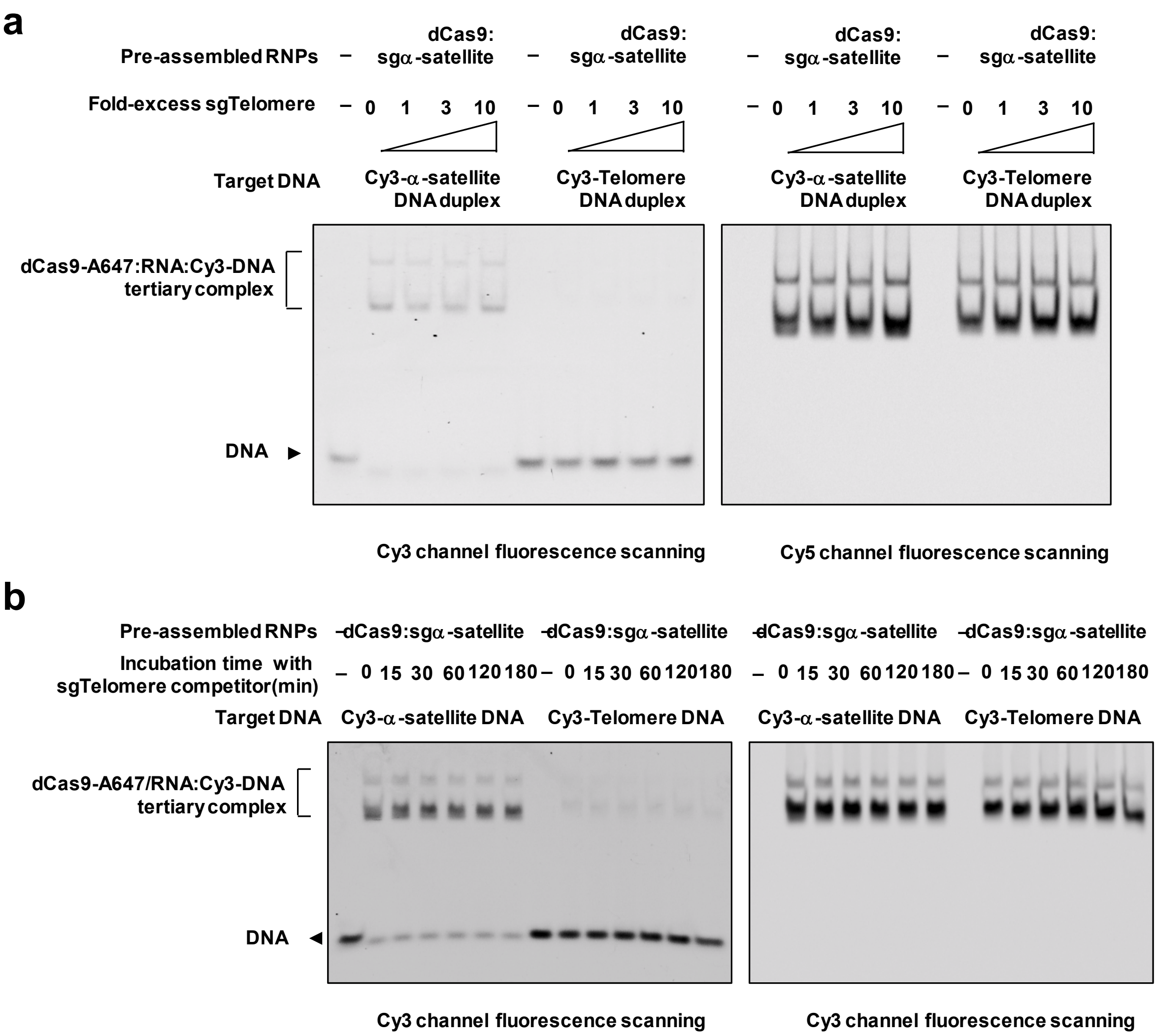

Supplementary Figure 4. Biochemical characterization of gRNA cross-talk/competition. (**a**) dCas9-Alexa 647 RNPs are assembled with a gRNA against α-satellite sequences (sgα-satellite) and incubated with 1-10-fold excess of a competitor gRNA (sgTelomere). Binding is only observed for the Cy3-α-satellite DNA target duplex but not for the Cy3-Telomere DNA duplex. (**b**) Time course of competition experiment. Very little binding to the Cy3-Telomere DNA duplex is observed, even after 180 minute-long incubation.

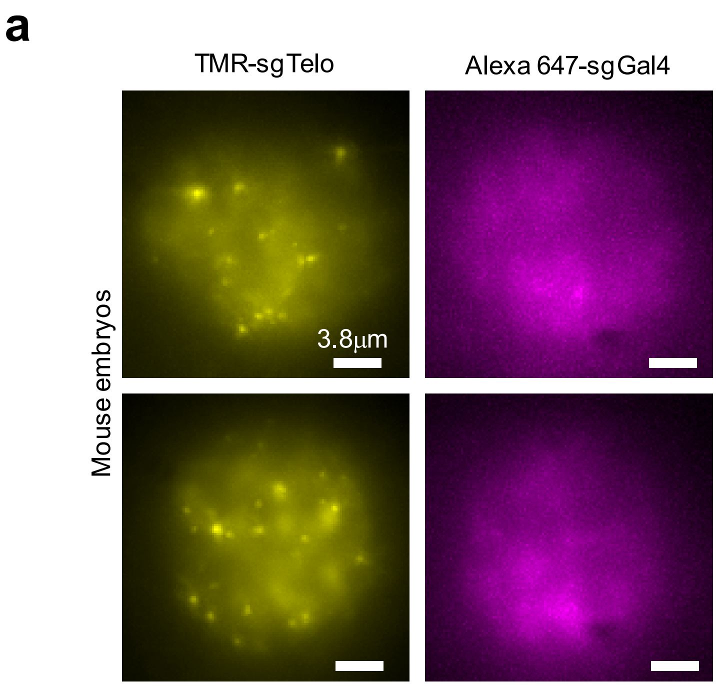

Supplementary Figure 5. Specificity of fluorescent dCas9-gRNA RNPs delivered in live 2-cell-stage mouse embryos. (**a**) Nuclear puncta are observed for dCas9-TMR assembled with a targeting gRNA (sgTelo), but no puncta are observed for dCas9-Alexa 647 assembled with a non-targeting gRNA (sgGal4).

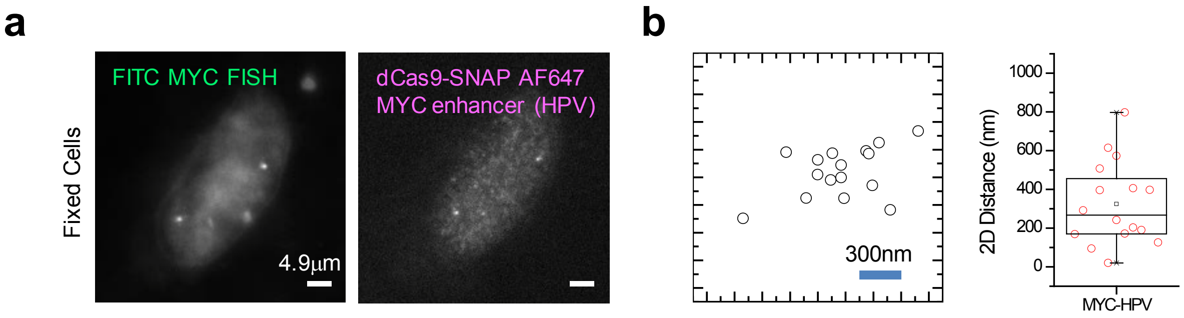

Supplementary Figure 6. Validation of dCas9-HPV tagging using DNA FISH in fixed HeLa cells. (**a**) Two-color imaging of FITC MYC FISH probes and dCas9-Alexa 647-HPV RNPs. (**b**) Scatter plot and 2D distances between HPV loci vs. the centroid of the *MYC* FISH probes.

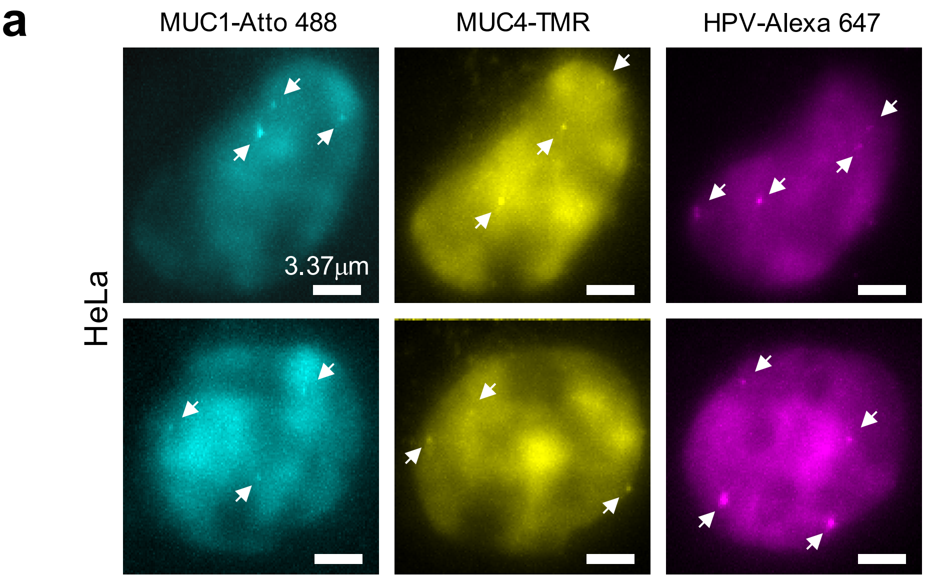

Supplementary Figure 7. Multiplex imaging of 3 genes. (**a**) Atto 488-MUC1, TMR-MUC4 and Alexa 647-HPV RNPs co-delivered in live HeLa cells. Arrows show nuclear puncta.

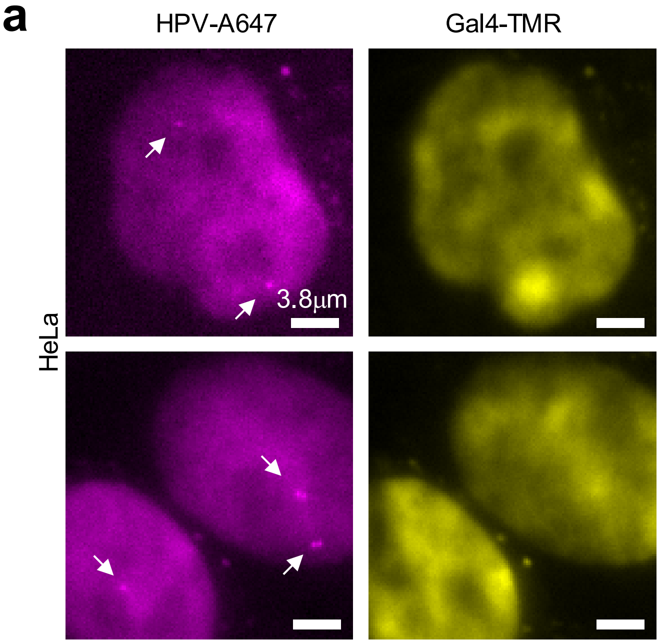

Supplementary Figure 8. Control experiments for two-color imaging of genomic loci in live HeLa cells. (**a**) Imaging of co-delivered Alexa 647-HPV and Gal4-TMR RNPs. Arrows indicate nuclear puncta for Alexa 647-HPV. No discernible nuclear puncta are seen for Gal4-TMR RNPs.

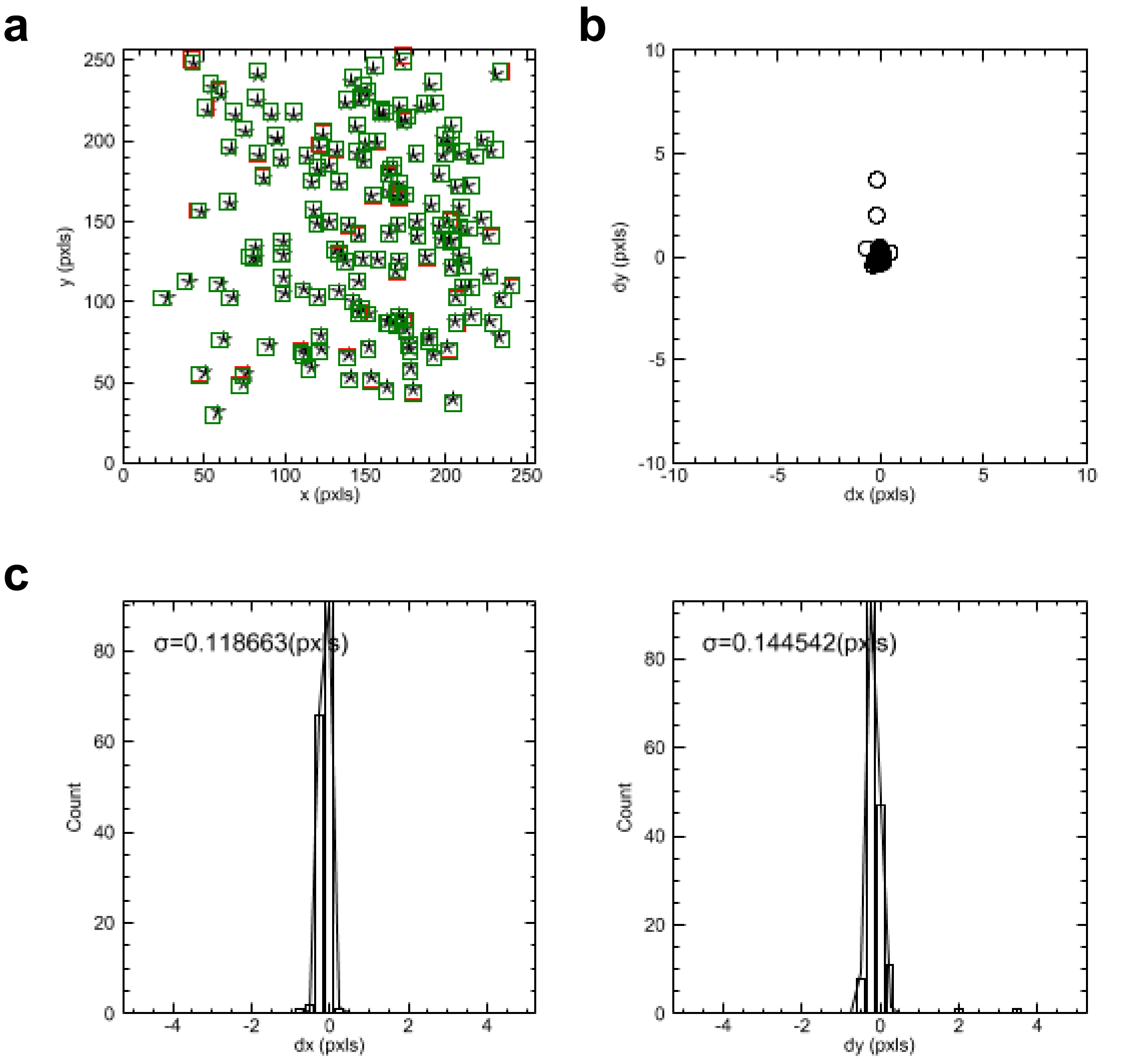

Supplementary Figure 9. Two-color registration. (**a**) Scatter plot of coordinates of 100 nm Tetraspek beads. (**b**) Scatter plot and (**c**) histograms of relative coordinates between TMR and Alexa 647 channel.

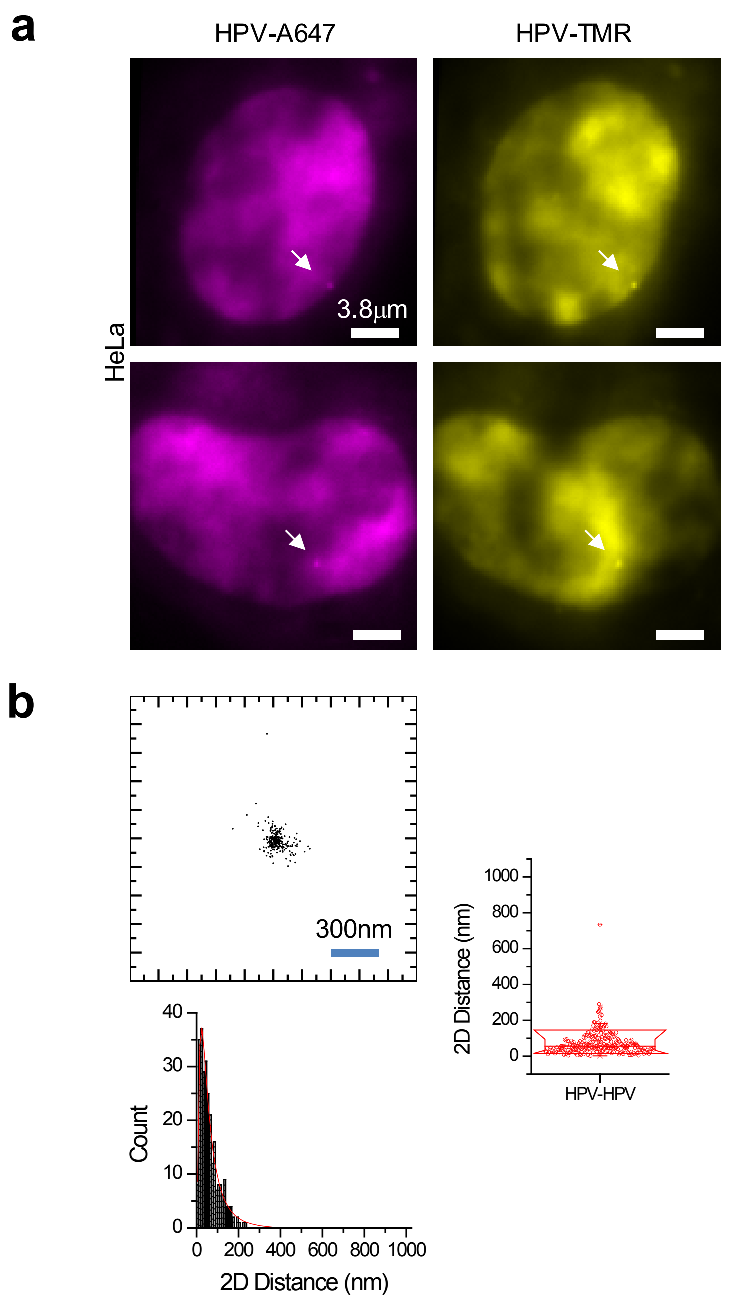

Supplementary Figure 10. Characterization of two-color 2D distance measurement precision in live cells. (**a**) Two-color imaging of HPV integration site, simultaneously in two colors (Alexa 647 and TMR) using co-delivery of preassembled RNPs in live HeLa cells. (**b**) 2D scatter plot of relative positions and statistics (histogram and box plot) of 2D distances between Alexa 647-HPV and TMR-HPV. 2D distance is 61±45 nm (mean±S.D.; *n*=271).

Supplementary Material

DNA Sequences

Sequence of the dCas9-SNAP/CLIP Proteins

dCas9-SNAP fusion protein:

AGATCT*CCCAAAAAGAAGAGGAAAGTG***ATGGATAAGAAATACTCAATAGGCTTAGCTATCGGCA**
CAAATAGCGTCGGATGGGCGGTGATCACTGATGAATATAAGGTTCCGTCTAAAAAGTTCAAGG
TTCTGGGAAATACAGACCGCCACAGTATCAAAAAAAATCTTATAGGGGCTCTTTTATTTGACAG
TGGAGAGACAGCGGAAGCGACTCGTCTCAAACGGACAGCTCGTAGAAGGTATACACGTCGGA
AGAATCGTATTTGTTATCTACAGGAGATTTTTTCAAATGAGATGGCGAAAGTAGATGATAGTTT
CTTTCATCGACTTGAAGAGTCTTTTTTGGTGGAAGAAGACAAGAAGCATGAACGTCATCCTATT
TTTGGAAATATAGTAGATGAAGTTGCTTATCATGAGAAATATCCAACTATCTATCATCTGCGAA
AAAAATTGGTAGATTCTACTGATAAAGCGGATTTGCGCTTAATCTATTTGGCCTTAGCGCATAT
GATTAAGTTTCGTGGTCATTTTTTGATTGAGGGAGATTTAAATCCTGATAATAGTGATGTGGACA
AACTATTTATCCAGTTGGTACAAACCTACAATCAATTATTTGAAGAAAACCCTATTAACGCAAG
TGGAGTAGATGCTAAAGCGATTCTTTCTGCACGATTGAGTAAATCAAGACGATTAGAAAATCTC
ATTGCTCAGCTCCCCGGTGAGAAGAAAAATGGCTTATTTGGGAATCTCATTGCTTTGTCATTGG
GTTTGACCCCTAATTTTAAATCAAATTTTGATTTGGCAGAAGATGCTAAATTACAGCTTTCAAAA
GATACTTACGATGATGATTTAGATAATTTATTGGCGCAAATTGGAGATCAATATGCTGATTTGTT
TTTGGCAGCTAAGAATTTATCAGATGCTATTTTACTTTCAGATATCCTAAGAGTAAATACTGAAA
TAACTAAGGCTCCCCTATCAGCTTCAATGATTAAACGCTACGATGAACATCATCAAGACTTGAC
TCTTTTAAAAGCTTTAGTTCGACAACAACTTCCAGAAAAGTATAAAGAAATCTTTTTTGATCAAT
CAAAAAACGGATATGCAGGTTATATTGATGGGGGAGCTAGCCAAGAAGAATTTTATAAATTTAT
CAAACCAATTTTAGAAAAAATGGATGGTACTGAGGAATTATTGGTGAAACTAAATCGTGAAGAT
TTGCTGCGCAAGCAACGGACCTTTGACAACGGCTCTATTCCCCATCAAATTCACTTGGGTGAG
CTGCATGCTATTTTGAGAAGACAAGAAGACTTTTATCCATTTTTAAAAGACAATCGTGAGAAGA
TTGAAAAAATCTTGACTTTTCGAATTCCTTATTATGTTGGTCCATTGGCGCGTGGCAATAGTCGT
TTTGCATGGATGACTCGGAAGTCTGAAGAAACAATTACCCCATGGAATTTTGAAGAAGTTGTCG
ATAAAGGTGCTTCAGCTCAATCATTTATTGAACGCATGACAAACTTTGATAAAAATCTTCCAAA
TGAAAAAGTACTACCAAAACATAGTTTGCTTTATGAGTATTTTACGGTTTATAACGAATTGACAA
AGGTCAAATATGTTACTGAAGGAATGCGAAAACCAGCATTTCTTTCAGGTGAACAGAAGAAAG
CCATTGTTGATTTACTCTTCAAAACAAATCGAAAAGTAACCGTTAAGCAATTAAAAGAAGATTA
TTTCAAAAAAATAGAATGTTTTGATAGTGTTGAAATTTCAGGAGTTGAAGATAGATTTAATGCTT
CATTAGGTACCTACCATGATTTGCTAAAAATTATTAAAGATAAAGATTTTTTGGATAATGAAGAA
AATGAAGATATCTTAGAGGATATTGTTTTAACATTGACCTTATTTGAAGATAGGGAGATGATTG
AGGAAAGACTTAAAACATATGCTCACCTCTTTGATGATAAGGTGATGAAACAGCTTAAACGTC
GCCGTTATACTGGTTGGGGACGTTTGTCTCGAAAATTGATTAATGGTATTAGGGATAAGCAATC
TGGCAAAACAATATTAGATTTTTTGAAATCAGATGGTTTTGCCAATCGCAATTTTATGCAGCTGA

TCCATGATGATAGTTTGACATTTAAAGAAGACATTCAAAAAGCACAAGTGTCTGGACAAGGCG
ATAGTTTACATGAACATATTGCAAATTTAGCTGGTAGCCCTGCTATTAAAAAAGGTATTTTACAG
ACTGTAAAAGTTGTTGATGAATTGGTCAAAGTAATGGGGCGGCATAAGCCAGAAAATATCGTT
ATTGAAATGGCACGTGAAAATCAGACAACTCAAAAGGGCCAGAAAAATTCGCGAGAGCGTAT
GAAACGAATCGAAGAAGGTATCAAAGAATTAGGAAGTCAGATTCTTAAAGAGCATCCTGTTGA
AAATACTCAATTGCAAAATGAAAAGCTCTATCTCTATTATCTCCAAAATGGAAGAGACATGTAT
GTGGACCAAGAATTAGATATTAATCGTTTAAGTGATTATGATGTCGATGCCATTGTTCCACAAA
GTTTCCTTAAAGACGATTCAATAGACAATAAGGTCTTAACGCGTTCTGATAAAAATCGTGGTAA
ATCGGATAACGTTCCAAGTGAAGAAGTAGTCAAAAAGATGAAAAACTATTGGAGACAACTTCT
AAACGCCAAGTTAATCACTCAACGTAAGTTTGATAATTTAACGAAAGCTGAACGTGGAGGTTTG
AGTGAACTTGATAAAGCTGGTTTTATCAAACGCCAATTGGTTGAAACTCGCCAAATCACTAAGC
ATGTGGCACAAATTTTGGATAGTCGCATGAATACTAAATACGATGAAAATGATAAACTTATTCG
AGAGGTTAAAGTGATTACCTTAAAATCTAAATTAGTTTCTGACTTCCGAAAAGATTTCCAATTCT
ATAAAGTACGTGAGATTAACAATTACCATCATGCCCATGATGCGTATCTAAATGCCGTCGTTGG
AACTGCTTTGATTAAGAAATATCCAAAACTTGAATCGGAGTTTGTCTATGGTGATTATAAAGTTT
ATGATGTTCGTAAAATGATTGCTAAGTCTGAGCAAGAAATAGGCAAAGCAACCGCAAAATATT
TCTTTTACTCTAATATCATGAACTTCTTCAAAACAGAAATTACACTTGCAAATGGAGAGATTCGC
AAACGCCCTCTAATCGAAACTAATGGGGAAACTGGAGAAATTGTCTGGGATAAAGGGCGAGA
TTTTGCCACAGTGCGCAAAGTATTGTCCATGCCCCAAGTCAATATTGTCAAGAAAACAGAAGTA
CAGACAGGCGGATTCTCCAAGGAGTCAATTTTACCAAAAAGAAATTCGGACAAGCTTATTGCT
CGTAAAAAAGACTGGGATCCAAAAAAATATGGTGGTTTTGATAGTCCAACGGTAGCTTATTCA
GTCCTAGTGGTTGCTAAGGTGGAAAAAGGGAAATCGAAGAAGTTAAAATCCGTTAAAGAGTTA
CTAGGGATCACAATTATGGAAAGAAGTTCCTTTGAAAAAAATCCGATTGACTTTTTAGAAGCTA
AAGGATATAAGGAAGTTAAAAAAGACTTAATCATTAAACTACCTAAATATAGTCTTTTTGAGTTA
GAAAACGGTCGTAAACGGATGCTGGCTAGTGCCGGAGAATTACAAAAAGGAAATGAGCTGGC
TCTGCCAAGCAAATATGTGAATTTTTTATATTTAGCTAGTCATTATGAAAAGTTGAAGGGTAGTC
CAGAAGATAACGAACAAAAACAATTGTTTGTGGAGCAGCATAAGCATTATTTAGATGAGATTAT
TGAGCAAATCAGTGAATTTTCTAAGCGTGTTATTTTAGCAGATGCCAATTTAGATAAAGTTCTTA
GTGCATATAACAAACATAGAGACAAACCAATACGTGAACAAGCAGAAAATATTATTCATTTATT
TACGTTGACGAATCTTGGAGCTCCCGCTGCTTTTAAATATTTTGATACAACAATTGATCGTAAAC
GATATACGTCTACAAAAGAAGTTTTAGATGCCACTCTTATCCATCAATCCATCACTGGTCTTTAT
GAAACACGCATTGATTTGAGTCAGCTAGGAGGTGACGAGGGAGCT*CCCAAGAAAAAGCGCAAG*
*GTA*GTCGACGGTGGTTCTGGTATGGACAAAGACTGCGAAATGAAGCGCACCACCCTGGATAGCC
CTCTGGGCAAGCTGGAACTGTCTGGGTGCGAACAGGGCCTGCACCGTATCATCTTCCTGGGCAA
AGGAACATCTGCCGCCGACGCCGTGGAAGTGCCTGCCCCAGCCGCCGTGCTGGGCGGACCAG
AGCCACTGATGCAGGCCACCGCCTGGCTCAACGCCTACTTTCACCAGCCTGAGGCCATCGAGG
AGTTCCCTGTGCCAGCCCTGCACCACCCAGTGTTCCAGCAGGAGAGCTTTACCCGCCAGGTGCT
GTGGAAACTGCTGAAAGTGGTGAAGTTCGGAGAGGTCATCAGCTACAGCCACCTGGCCGCCCT
GGCCGGCAATCCCGCCGCCACCGCCGCCGTGAAAACCGCCCTGAGCGGAAATCCCGTGCCCAT
TCTGATCCCCTGCCACCGGGTGGTGCAGGGCGACCTGGACGTGGGGGGCTACGAGGGCGGGC

TCGCCGTGAAAGAGTGGCTGCTGGCCCACGAGGGCCACAGACTGGGCAAGCCTGGGCTGGGT
CTCGAGGGTGGTTCTGGTCTGGAAGTTCTGTTCCAGGGGCCCCATCATCACCATCACCACCATC
ATCACCATTAA

The CLIP sequence in pD451-SR-dCas9-CLIP plasmid is:

ATGGACAAAGACTGCGAAATGAAGCGCACCACCCTGGATAGCCCTCTGGGCAAGCTGGAACTGT
CTGGGTGCGAACAGGGCCTGCACCGTATCATCTTCCTGGGCAAAGGAACATCTGCCGCCGACG
CCGTGGAAGTGCCTGCCCCAGCCGCCGTGCTGGGCGGACCAGAGCCACTGATCCAGGCCACC
GCCTGGCTCAACGCCTACTTTCACCAGCCTGAGGCCATCGAGGAGTTCCCTGTGCCAGCCCTGC
ACCACCCAGTGTTCCAGCAGGAGAGCTTTACCCGCCAGGTGCTGTGGAAACTGCTGAAAGTGGT
GAAGTTCGGAGAGGTCATCAGCGAGAGCCACCTGGCCGCCCTGGTGGGCAATCCCGCCGCCAC
CGCCGCCGTGAACACCGCCCTGGACGGAAATCCCGTGCCCATTCTGATCCCCTGCCACCGGGT
GGTGCAGGGCGACAGCGACGTGGGGCCCTACCTGGGCGGGCTCGCCGTGAAAGAGTGGCTGC
TGGCCCACGAGGGCCACAGACTGGGCAAGCCTGGGCTGGGT

Notes: *italics* indicate nuclear localization signal (NLS) sequence; **bold** text is the sequence of dCas9; underlined text is the SNAPf sequence; red text is GGSG linker sequence; blue text is HRV 3C site and purple text is His_{10} affinity tag.

Synthetic sgTelomere DNA for cloning into pLVX-BFP plasmid:

ggatcc**GTTAGGGTTAGGGTTAGGGTTA**GTTTAAGAGCTATGCTGGAAACAGCATAGCAAGTTTAA
ATAAGGCTAGTCCGTTATCAACTTGAAAAAGTGGCACCGAGTCGGTGCTTTTTTTacgcgtgaattc

Note: bold text is the sequence of sgTelomere and underlined text is the sgRNA scaffold.

Supplementary Tables

Supplementary Table 1. Primers for *in vitro* T7 transcribed sgGAL4, sgTelomere, sg□satellite, sgMUC4 and sgMUC1

Primer name	Sequence 5’-3’
Common REV	AAAAAAAGCACCGACTCGGTGCCAC
sgGAL4 FWD	GAAAT**TAATACGACTCACTATAG**<u>GTTGGAGCACTGTCCTCCGAACGT</u>GTTTAAG
sgTelomere FWD	GAAAT**TAATACGACTCACTATAG**<u>GTTAGGGTTAGGGTTAGGGTTA</u>G
sgα-satellite FWD	GAAAT**TAATACGACTCACTATAG**<u>GTAGAATCTGCAAGTGGATATT</u>GTT
sgMUC4 E3 FWD	GAAAT**TAATACGACTCACTATAG**<u>GTGGCGTGACCTGTGGATGCTG</u>GTTTAAGAGCTATGCTGGAAACA
sgMUC1 E3 FWD	GAAAT**TAATACGACTCACTATAG**<u>GTCCGGGGCCGAGGTGACACCGT</u>GTTTAAGAGCTATGCTGGAAACA

Bold, T7 promoter; <u>underlined</u>, sequence of sgRNA target; FWD, forward primer; REV: reverse primer.

Supplementary Table 2. Primers for *in vitro* T7 transcribed gRNAs targeting the HPV-18 integration site in HeLa cells

Primer name	Sequence 5’-3’
sgHPV FWD1	GAAAT**TAATACGACTCACTATAG**<u>ATATTTGTCAAATGCCAAAT</u>GTTTAAGAGCTATGCTGGAAACA
sgHPV FWD2	GAAAT**TAATACGACTCACTATAG**<u>GAATGTTTAACTTCTAGGCC</u>GTTTAAGAGCTATGCTGGAAACA
sgHPV FWD3	GAAAT**TAATACGACTCACTATAG**<u>GAATGCCTTCAAAGAACAGCA</u>GTTTAAGAGCTATGCTGGAAACA
sgHPV FWD4	GAAAT**TAATACGACTCACTATAG**<u>GCAATGCTTAACAC</u>

	GGCAGCGTTTAAGAGCTATGCTGGAAACA
sgHPV FWD5	GAAAT**TAATACGACTCACTATAG**GCATTGCTAATCTAGAAGAAGTTTAAGAGCTATGCTGGAAACA
sgHPV FWD6	GAAAT**TAATACGACTCACTATAG**GAAAAGAAGCAGAGGTATGCGTGTTTAAGAGCTATGCTGGAAACA
sgHPV FWD7	GAAAT**TAATACGACTCACTATAG**GTTCAATTGGTATGATTTAAGTTTAAGAGCTATGCTGGAAACA
sgHPV FWD8	GAAAT**TAATACGACTCACTATAG**ATAAGGGCCACATAATGGAGGTTTAAGAGCTATGCTGGAAACA
sgHPV FWD9	GAAAT**TAATACGACTCACTATAG**GACATTAATCTTAAGTATCCAGTTTAAGAGCTATGCTGGAAACA
sgHPV FWD10	GAAAT**TAATACGACTCACTATAG**GTGTGATGGGAGCCATGTGGTTTAAGAGCTATGCTGGAAACA
sgHPV FWD11	GAAAT**TAATACGACTCACTATAG**GGCAACTTCAGGCCATAGTCACGTTTAAGAGCTATGCTGGAAACA
sgHPV FWD12	GAAAT**TAATACGACTCACTATAG**GCTAGACTCATTCATGCATTCGTTTAAGAGCTATGCTGGAAACA
sgHPV FWD13	GAAAT**TAATACGACTCACTATAG**GTTCAATCATTTAGAGAAGAGTTTAAGAGCTATGCTGGAAACA
sgHPV FWD14	GAAAT**TAATACGACTCACTATAG**AAGGACTTCTTCATGTACCCGTTTAAGAGCTATGCTGGAAACA
sgHPV FWD15	GAAAT**TAATACGACTCACTATAG**ATACACCGAGGGAAAAACCAGTTTAAGAGCTATGCTGGAAACA
sgHPV FWD16	GAAAT**TAATACGACTCACTATAG**GTACCTGGCCAAGATTAATTGTTTAAGAGCTATGCTGGAAACA
sgHPV FWD17	GAAAT**TAATACGACTCACTATAG**GTCTGTGCAAAAAAAGTGCAGGTTTAAGAGCTATGCTGGAAACA
sgHPV FWD18	GAAAT**TAATACGACTCACTATAG**GAAGTTCTCCTCCAAGGAAGGTTTAAGAGCTATGCTGGAAACA
sgHPV FWD19	GAAAT**TAATACGACTCACTATAG**GGATAATTTGGGTCTCAAAAGTTTAAGAGCTATGCTGGAAACA

sgHPV FWD20	GAAAT**TAATACGACTCACTATAG**GCTTCTGGGGAAGAGGAGTAGTTTAAGAGCTATGCTGGAAACA
sgHPV FWD21	GAAAT**TAATACGACTCACTATAG**ATAAGATAGGGACCTAGACAGTTTAAGAGCTATGCTGGAAACA
sgHPV FWD22	GAAAT**TAATACGACTCACTATAG**GAGAAATGTTATTGAAACGTTTAAGAGCTATGCTGGAAACA
sgHPV FWD23	GAAAT**TAATACGACTCACTATAG**GTGCAGAGTGACACAGTATTGTTTAAGAGCTATGCTGGAAACA
sgHPV FWD24	GAAAT**TAATACGACTCACTATAG**GATGCCATTATAGACAAGAACGTTTAAGAGCTATGCTGGAAACA
sgHPV FWD25	GAAAT**TAATACGACTCACTATAG**GTAGAACAGACAGGACTACAGTTTAAGAGCTATGCTGGAAACA
sgHPV FWD26	GAAAT**TAATACGACTCACTATAG**GCCAACTAGCCTCAAATTAAAGCAGTTTAAGAGCTATGCTGGAAACA
sgHPV FWD27	GAAAT**TAATACGACTCACTATAG**GCCCCAAATCCAGGTTCCCCGTTTAAGAGCTATGCTGGAAACA
sgHPV FWD28	GAAAT**TAATACGACTCACTATAG**GCTTACGGATCTACTTCTAATGTTTAAGAGCTATGCTGGAAACA
sgHPV FWD29	GAAAT**TAATACGACTCACTATAG**GCTCCACCAAGATGGGAAGGAGTTTAAGAGCTATGCTGGAAACA
sgHPV FWD30	GAAAT**TAATACGACTCACTATAG**GCCAAGATATAGTTGGCCAGGTTTAAGAGCTATGCTGGAAACA
sgHPV FWD31	GAAAT**TAATACGACTCACTATAG**ACACAGCTATCAGAGCAAGAGTTTAAGAGCTATGCTGGAAACA
sgHPV FWD32	GAAAT**TAATACGACTCACTATAG**GACTCATTCTTTGGCTAGTTTAAGAGCTATGCTGGAAACA
sgHPV FWD33	GAAAT**TAATACGACTCACTATAG**GTAAAGAATAAACAATAGATGTTTAAGAGCTATGCTGGAAACA
sgHPV FWD34	GAAAT**TAATACGACTCACTATAG**AGGAACAAAGGAATCGAGGGGTTTAAGAGCTATGCTGGAAACA
sgHPV FWD35	GAAAT**TAATACGACTCACTATAG**GGAACAAAAGAAC

	AAAAAAGGTTTAAGAGCTATGCTGGAAACA
sgHPV FWD36	GAAAT**TAATACGACTCACTATAG**GTTTCCCTATGAAAATGGCAAGTTTAAGAGCTATGCTGGAAACA

Bold, T7 promoter；underlined, sequence of sgRNA target; FWD, forward primer.

Supplementary Table 3. Primers for *in vitro* T7 transcribed gRNAs targeting *MYC*

Primer name	Sequence 5’-3’
sgMYC FWD1	GAAAT**TAATACGACTCACTATAG**GACCAAGTCTTGCTTACTGGTGTTTAAGAGCTATGCTGGAAACA
sgMYC FWD2	GAAAT**TAATACGACTCACTATAG**GCTAAAGTACTCAAAGCAGGTTTAAGAGCTATGCTGGAAACA
sgMYC FWD3	GAAAT**TAATACGACTCACTATAG**GTTTAGGCAGGGCGAGGGGGGTTTAAGAGCTATGCTGGAAACA
sgMYC FWD4	GAAAT**TAATACGACTCACTATAG**GCTTGGATAACTTCTTGCAGGTTTAAGAGCTATGCTGGAAACA
sgMYC FWD5	GAAAT**TAATACGACTCACTATAG**GATGCCAAAGTCAGGCTAGTTTAAGAGCTATGCTGGAAACA
sgMYC FWD6	GAAAT**TAATACGACTCACTATAG**GAACAAAGAATCTGTTATAGTTTAAGAGCTATGCTGGAAACA
sgMYC FWD7	GAAAT**TAATACGACTCACTATAG**GTTAATAAAAGCTGACTTCACGTTTAAGAGCTATGCTGGAAACA
sgMYC FWD8	GAAAT**TAATACGACTCACTATAG**GGAACTCATTGAAGTTCGTTTAAGAGCTATGCTGGAAACA
sgMYC FWD9	GAAAT**TAATACGACTCACTATAG**ATAAAACTGAAATAATTAATGTTTAAGAGCTATGCTGGAAACA
sgMYC FWD10	GAAAT**TAATACGACTCACTATAG**ACTCACCCAAAAAACCAGCTGTTTAAGAGCTATGCTGGAAACA
sgMYC FWD11	GAAAT**TAATACGACTCACTATAG**GAATGGTAAAGGACAGGATGTTTAAGAGCTATGCTGGAAACA

sgMYC FWD12	GAAAT**TAATACGACTCACTATAG**GCTTCACCAGAGAAGCAGAGTTTAAGAGCTATGCTGGAAACA
sgMYC FWD13	GAAAT**TAATACGACTCACTATAG**GTTGATTTGGTAATAGGCAGGTTTAAGAGCTATGCTGGAAACA
sgMYC FWD14	GAAAT**TAATACGACTCACTATAG**GACATTGGAAACTGGCCAGTTTAAGAGCTATGCTGGAAACA
sgMYC FWD15	GAAAT**TAATACGACTCACTATAG**GCACCCTCCAGAGCTGCAGCGGTTTAAGAGCTATGCTGGAAACA
sgMYC FWD16	GAAAT**TAATACGACTCACTATAG**GTTCTAAATACATTTGGACGGGTTTAAGAGCTATGCTGGAAACA
sgMYC FWD17	GAAAT**TAATACGACTCACTATAG**GTCCCAGTCTGCAAAATAAAGGTTTAAGAGCTATGCTGGAAACA
sgMYC FWD18	GAAAT**TAATACGACTCACTATAG**GGCTGTACCCCCAGTGATAGTTTAAGAGCTATGCTGGAAACA
sgMYC FWD19	GAAAT**TAATACGACTCACTATAG**ACTGTTAACCTTAACATCAACGTTTAAGAGCTATGCTGGAAACA
sgMYC FWD20	GAAAT**TAATACGACTCACTATAG**GGTGCACAGGAGAAGAAATGTTTAAGAGCTATGCTGGAAACA
sgMYC FWD21	GAAAT**TAATACGACTCACTATAG**ACAAATCCTTGTCCTCCAAGGTTTAAGAGCTATGCTGGAAACA
sgMYC FWD22	GAAAT**TAATACGACTCACTATAG**GTCCTGGGGCATAATGCCAAGGTTTAAGAGCTATGCTGGAAACA
sgMYC FWD23	GAAAT**TAATACGACTCACTATAG**GACCAGAGCAAATTTCCTTAGTTTAAGAGCTATGCTGGAAACA
sgMYC FWD24	GAAAT**TAATACGACTCACTATAG**GCTTCCTTCTTCAATTCAGATGTTTAAGAGCTATGCTGGAAACA
sgMYC FWD25	GAAAT**TAATACGACTCACTATAG**AGAGGGATTATAAAGTTGCGGTTTAAGAGCTATGCTGGAAACA
sgMYC FWD26	GAAAT**TAATACGACTCACTATAG**ACACTGTTGTTGAAGTGGTTTAAGAGCTATGCTGGAAACA
sgMYC FWD27	GAAAT**TAATACGACTCACTATAG**ATCGTTGGAGCAAGGGTGACG

GTTTAAGAGCTATGCTGGAAACA

sgMYC FWD28 GAAAT**TAATACGACTCACTATAG**GCTTCGGTTCCATCAATGTTTAAGAGCTATGCTGGAAACA

sgMYC FWD29 GAAAT**TAATACGACTCACTATAG**GTTAATCGTATATAGAGAGAAGTTTAAGAGCTATGCTGGAAACA

sgMYC FWD30 GAAAT**TAATACGACTCACTATAG**GAGAGAGCTTCTGAGCTGAGTTTAAGAGCTATGCTGGAAACA

sgMYC FWD31 GAAAT**TAATACGACTCACTATAG**ACTCTGTGAATGGCACCTTGGTTTAAGAGCTATGCTGGAAACA

sgMYC FWD32 GAAAT**TAATACGACTCACTATAG**AGAAGCTTCTGGGGTCAGTAGTTTAAGAGCTATGCTGGAAACA

sgMYC FWD33 GAAAT**TAATACGACTCACTATAG**GGGATGGTTTAAACTCTCAAGTTTAAGAGCTATGCTGGAAACA

sgMYC FWD34 GAAAT**TAATACGACTCACTATAG**GGATCATGAAAAGCTCCTACGTTTAAGAGCTATGCTGGAAACA

sgMYC FWD35 GAAAT**TAATACGACTCACTATAG**GGCAGGCTGGCAGCCTGAGTTTAAGAGCTATGCTGGAAACA

sgMYC FWD36 GAAAT**TAATACGACTCACTATAG**GTTTCCCAACACACTGCTGCGTTTAAGAGCTATGCTGGAAACA

sgMYC FWD37 GAAAT**TAATACGACTCACTATAG**AAAGCGTAAATCAACAACCGTTTAAGAGCTATGCTGGAAACA

sgMYC FWD38 GAAAT**TAATACGACTCACTATAG**GGTGTGGTCCTGGAAACCCAGTTTAAGAGCTATGCTGGAAACA

sgMYC FWD39 GAAAT**TAATACGACTCACTATAG**ATATGAATTCAGTGAAAGGGTTTAAGAGCTATGCTGGAAACA

sgMYC FWD40 GAAAT**TAATACGACTCACTATAG**AAAATTCTGGAAGTTCAGGAGTTTAAGAGCTATGCTGGAAACA

sgMYC FWD41 GAAAT**TAATACGACTCACTATAG**GCAAGAGTTGGAGACAGGAAGTTTAAGAGCTATGCTGGAAACA

sgMYC FWD42 GAAAT**TAATACGACTCACTATAG**AATACAAGCTTGTCAAGTTGGTTTAAGAGCTATGCTGGAAACA

sgMYC FWD43 GAAAT**TAATACGACTCACTATAG**GGTAAGAGAATGAAGTCAATGTTTAAGAGCTATGCTGGAAACA

sgMYC FWD44 GAAAT**TAATACGACTCACTATAG**ACATTCATTCACATTAACTATGTTTAAGAGCTATGCTGGAAACA

sgMYC FWD45 GAAAT**TAATACGACTCACTATAG**GAAGAAGAGAAAAAGGGTTTAAGAGCTATGCTGGAAACA

sgMYC FWD46 GAAAT**TAATACGACTCACTATAG**GGATTCTACCCTGAGAACAAGTTTAAGAGCTATGCTGGAAACA

sgMYC FWD47 GAAAT**TAATACGACTCACTATAG**GATTCTGTAAGATGATCATGTTTAAGAGCTATGCTGGAAACA

sgMYC FWD48 GAAAT**TAATACGACTCACTATAG**GGGCTGGGAGGAAAGGGTTTAAGAGCTATGCTGGAAACA

sgMYC FWD49 GAAAT**TAATACGACTCACTATAG**GAGAAGAGACAGTAAGTCTGGTTTAAGAGCTATGCTGGAAACA

sgMYC FWD50 GAAAT**TAATACGACTCACTATAG**GAAACTTGTGTGTCACTCAGAGGTTTAAGAGCTATGCTGGAAACA

sgMYC FWD51 GAAAT**TAATACGACTCACTATAG**GAAATAGGGTAAGATAAGGAGGGTTTAAGAGCTATGCTGGAAACA

sgMYC FWD52 GAAAT**TAATACGACTCACTATAG**ATTCTTCAGTTGACCCCCAGTTTAAGAGCTATGCTGGAAACA

sgMYC FWD53 GAAAT**TAATACGACTCACTATAG**GGAAAATTAATCAGTGTGCAGTTTAAGAGCTATGCTGGAAACA

sgMYC FWD54 GAAAT**TAATACGACTCACTATAG**GTCCATGTAGAAGACGTTACAGGTTTAAGAGCTATGCTGGAAACA

sgMYC FWD55 GAAAT**TAATACGACTCACTATAG**ATAAAAGTGAAAACAACCAAGTTTAAGAGCTATGCTGGAAACA

sgMYC FWD56 GAAAT**TAATACGACTCACTATAG**GCAGAGATCCCAAGCTAGTTTAAGAGCTATGCTGGAAACA

sgMYC FWD57 GAAAT**TAATACGACTCACTATAG**AAGCAAAAGTTTCTCTCCAAGTTTAAGAGCTATGCTGGAAACA

sgMYC FWD58 GAAAT**TAATACGACTCACTATAG**GCTGTATGTCCAACCACGCAA

GTTTAAGAGCTATGCTGGAAACA

sgMYC FWD59 GAAAT**TAATACGACTCACTATAG**GGCCAGTGAGCCAAAGTGGTTTAAGAGCTATGCTGGAAACA

sgMYC FWD60 GAAAT**TAATACGACTCACTATAG**GTTGAACCCCTTCAAAGGCTGTTTAAGAGCTATGCTGGAAACA

sgMYC FWD61 GAAAT**TAATACGACTCACTATAG**AGTAGCTACAAAGTAAGGCAGTTTAAGAGCTATGCTGGAAACA

sgMYC FWD62 GAAAT**TAATACGACTCACTATAG**ATAAAAGAAGGCCAAGCTGGGTTTAAGAGCTATGCTGGAAACA

sgMYC FWD63 GAAAT**TAATACGACTCACTATAG**GTCCATTCTGAGACCTTCTGCTGTTTAAGAGCTATGCTGGAAACA

sgMYC FWD64 GAAAT**TAATACGACTCACTATAG**GGATATGGAAGCTCTCGTTTAAGAGCTATGCTGGAAACA

sgMYC FWD65 GAAAT**TAATACGACTCACTATAG**GCAGTATACTAAAAGCCAGGGTTTAAGAGCTATGCTGGAAACA

sgMYC FWD66 GAAAT**TAATACGACTCACTATAG**GCATAGGAATGATAACAAAAAGTTTAAGAGCTATGCTGGAAACA

sgMYC FWD67 GAAAT**TAATACGACTCACTATAG**GTTCCAACGAGAGGAACACGTTTAAGAGCTATGCTGGAAACA

sgMYC FWD68 GAAAT**TAATACGACTCACTATAG**GATTAAAAACCAAGCTAGCCAGTTTAAGAGCTATGCTGGAAACA

sgMYC FWD69 GAAAT**TAATACGACTCACTATAG**GATAAATATCTATCTCCAGGTTTAAGAGCTATGCTGGAAACA

sgMYC FWD70 GAAAT**TAATACGACTCACTATAG**GACAGGCCGCACGTGACTTGAGTTTAAGAGCTATGCTGGAAACA

sgMYC FWD71 GAAAT**TAATACGACTCACTATAG**GGTGAGACTGAAGCCCCTCAGTTTAAGAGCTATGCTGGAAACA

sgMYC FWD72 GAAAT**TAATACGACTCACTATAG**GATGGAAAGCAACTGGACGGTTTAAGAGCTATGCTGGAAACA

sgMYC FWD73 GAAAT**TAATACGACTCACTATAG**GATGTTCAAAGAAGGTGTTGGTTTAAGAGCTATGCTGGAAACA

sgMYC FWD74	GAAAT**TAATACGACTCACTATAG**GTTCTCCCCAGAGACACAAAAGTTTAAGAGCTATGCTGGAAACA
sgMYC FWD75	GAAAT**TAATACGACTCACTATAG**GTCATATATTTTTATACTTGTTTAAGAGCTATGCTGGAAACA
sgMYC FWD76	GAAAT**TAATACGACTCACTATAG**ACACACTATTCTGTTCTGTGGTTTAAGAGCTATGCTGGAAACA
sgMYC FWD77	GAAAT**TAATACGACTCACTATAG**GCAGGTCTCCTGGAGGGCCGGTTTAAGAGCTATGCTGGAAACA
sgMYC FWD78	GAAAT**TAATACGACTCACTATAG**GCAGCAGCTCCAAATAACAGGTTTAAGAGCTATGCTGGAAACA
sgMYC FWD79	GAAAT**TAATACGACTCACTATAG**GCTGCTAGAGCAACAAGCAAGTTTAAGAGCTATGCTGGAAACA
sgMYC FWD80	GAAAT**TAATACGACTCACTATAG**GGTCTTCCAAAAAAAATTGGTTTAAGAGCTATGCTGGAAACA
sgMYC FWD81	GAAAT**TAATACGACTCACTATAG**AATACTGACCAGTCAGGTTTAAGAGCTATGCTGGAAACA
sgMYC FWD82	GAAAT**TAATACGACTCACTATAG**GCCAATGGACACGTATCACTTGTTTAAGAGCTATGCTGGAAACA
sgMYC FWD83	GAAAT**TAATACGACTCACTATAG**GACATTTGCTGGGTTGAAAAAGTTTAAGAGCTATGCTGGAAACA
sgMYC FWD84	GAAAT**TAATACGACTCACTATAG**ATGTCCTTTAACCTGGCTGGTTTAAGAGCTATGCTGGAAACA
sgMYC FWD85	GAAAT**TAATACGACTCACTATAG**AGGTGATGTCACCAGCCTGAGTTTAAGAGCTATGCTGGAAACA
sgMYC FWD86	GAAAT**TAATACGACTCACTATAG**ACAGGCATTATATCTGCCTGGTTTAAGAGCTATGCTGGAAACA
sgMYC FWD87	GAAAT**TAATACGACTCACTATAG**GGCGGATATACCACATCTTGTTTAAGAGCTATGCTGGAAACA
sgMYC FWD88	GAAAT**TAATACGACTCACTATAG**ACAATGGCAAAACCAAAAGTGTTTAAGAGCTATGCTGGAAACA
sgMYC FWD89	GAAAT**TAATACGACTCACTATAG**GCTAAATGAGTGCTCTCCACAG

TTTAAGAGCTATGCTGGAAACA

sgMYC FWD90 GAAAT**TAATACGACTCACTATAG**GTCAGCCTACAAGGCTCCTGCGTTTAAGAGCTATGCTGGAAACA

sgMYC FWD91 GAAAT**TAATACGACTCACTATAG**GTCCACTGCCAGAAGTCCTTAGTTTAAGAGCTATGCTGGAAACA

sgMYC FWD92 GAAAT**TAATACGACTCACTATAG**GTAAAAACCTACTTGACCAGTTTAAGAGCTATGCTGGAAACA

sgMYC FWD93 GAAAT**TAATACGACTCACTATAG**ACTAAAATGAGTATGCAATAGTTTAAGAGCTATGCTGGAAACA

sgMYC FWD94 GAAAT**TAATACGACTCACTATAG**GATCTCATAGAAAAAAAGTGTGAGTTTAAGAGCTATGCTGGAAACA

sgMYC FWD95 GAAAT**TAATACGACTCACTATAG**GATGAGTTTCTAAGACGTGGGTTTAAGAGCTATGCTGGAAACA

sgMYC FWD96 GAAAT**TAATACGACTCACTATAG**AATTCCAACAAACCCTAAAAGTTTAAGAGCTATGCTGGAAACA

sgMYC FWD97 GAAAT**TAATACGACTCACTATAG**GCCATTACCGGTTCTCCATAGTTTAAGAGCTATGCTGGAAACA

sgMYC FWD98 GAAAT**TAATACGACTCACTATAG**GGAGTTACTGGAGGAAAAAGGTTTAAGAGCTATGCTGGAAACA

sgMYC FWD99 GAAAT**TAATACGACTCACTATAG**AACCTGAAAGAATAACAAGGGTTTAAGAGCTATGCTGGAAACA

sgMYC FWD100 GAAAT**TAATACGACTCACTATAG**GCTGGAAACCTTGCACCTGTTTAAGAGCTATGCTGGAAACA

sgMYC FWD101 GAAAT**TAATACGACTCACTATAG**ATCGCGCCTGGATGTCAACGAGTTTAAGAGCTATGCTGGAAACA

sgMYC FWD102 GAAAT**TAATACGACTCACTATAG**GTACTTTCGCAAACCTGAACGGTTTAAGAGCTATGCTGGAAACA

sgMYC FWD103 GAAAT**TAATACGACTCACTATAG**AGGCCTTTGCCGCAAACGCGGTTTAAGAGCTATGCTGGAAACA

sgMYC FWD104 GAAAT**TAATACGACTCACTATAG**GCTGAATTGTGCAGTGCATGTTTAAGAGCTATGCTGGAAACA

sgMYC FWD105 GAAAT**TAATACGACTCACTATAG**GAACGCTGAGCTGCAAACTCAAGTTTAAGAGCTATGCTGGAAACA

sgMYC FWD106 GAAAT**TAATACGACTCACTATAG**GCATGTACGCTGTTCAAGATGTTTAAGAGCTATGCTGGAAACA

sgMYC FWD107 GAAAT**TAATACGACTCACTATAG**GCAAAAGAGAAAACAATTCGGGTTTAAGAGCTATGCTGGAAACA

sgMYC FWD108 GAAAT**TAATACGACTCACTATAG**ATCCTTGGTCCCTCACCCAAGTTTAAGAGCTATGCTGGAAACA

sgMYC FWD109 GAAAT**TAATACGACTCACTATAG**GCACAAAATAAAAAATCCCGAGTTTAAGAGCTATGCTGGAAACA

sgMYC FWD110 GAAAT**TAATACGACTCACTATAG**GAGCAAACAAATCATGTGTGGTTTAAGAGCTATGCTGGAAACA

sgMYC FWD111 GAAAT**TAATACGACTCACTATAG**GTGAATACACGTTTGCGTTTAAGAGCTATGCTGGAAACA

sgMYC FWD112 GAAAT**TAATACGACTCACTATAG**GTGAACTAGGAAATTAATGCCGTTTAAGAGCTATGCTGGAAACA

sgMYC FWD113 GAAAT**TAATACGACTCACTATAG**GCCCCCCCCCAAAAAAAGGCAGTTTAAGAGCTATGCTGGAAACA

sgMYC FWD114 GAAAT**TAATACGACTCACTATAG**ATCGATTCTGATCAAAGAAGGTTTAAGAGCTATGCTGGAAACA

sgMYC FWD115 GAAAT**TAATACGACTCACTATAG**GACCGCATTTCCAATAATAAAAGTTTAAGAGCTATGCTGGAAACA

sgMYC FWD116 GAAAT**TAATACGACTCACTATAG**GTTAAACGTCCGGTTTGTCCGGTTTAAGAGCTATGCTGGAAACA

sgMYC FWD117 GAAAT**TAATACGACTCACTATAG**AGAGCTTGTGGACCGAGCCGGTTTAAGAGCTATGCTGGAAACA

sgMYC FWD118 GAAAT**TAATACGACTCACTATAG**GTAGACGGGAGAATATGGGAGGTTTAAGAGCTATGCTGGAAACA

sgMYC FWD119 GAAAT**TAATACGACTCACTATAG**GTTGCAAACCGGCGCCACAGTTTAAGAGCTATGCTGGAAACA

sgMYC FWD120 GAAAT**TAATACGACTCACTATAG**GAGAAATTGGGAACTCCGTGG

TTTAAGAGCTATGCTGGAAACA

sgMYC FWD121 GAAAT**TAATACGACTCACTATAG**<u>GCTGGGCTAGGGCGAGAGGG</u>
GTTTAAGAGCTATGCTGGAAACA

sgMYC FWD122 GAAAT**TAATACGACTCACTATAG**<u>GCTAAACAGACGCCTCCCGCA</u>
GTTTAAGAGCTATGCTGGAAACA

sgMYC FWD123 GAAAT**TAATACGACTCACTATAG**<u>GGGGGACTCAGTCTGGG</u>GTTT
AAGAGCTATGCTGGAAACA

sgMYC FWD124 GAAAT**TAATACGACTCACTATAG**<u>GACTCCCCCAACAAATGCAA</u>
GTTTAAGAGCTATGCTGGAAACA

sgMYC FWD125 GAAAT**TAATACGACTCACTATAG**<u>ACGCGCTCTCCAAGTATACG</u>G
TTTAAGAGCTATGCTGGAAACA

sgMYC FWD126 GAAAT**TAATACGACTCACTATAG**<u>GGAATGATAGAGGCATAAGG</u>G
TTTAAGAGCTATGCTGGAAACA

sgMYC FWD127 GAAAT**TAATACGACTCACTATAG**<u>GGGCGCGCGTTCAGAGCGT</u>GT
TTAAGAGCTATGCTGGAAACA

sgMYC FWD128 GAAAT**TAATACGACTCACTATAG**<u>GGGACTCTTGATCAAAGCG</u>GT
TTAAGAGCTATGCTGGAAACA

sgMYC FWD129 GAAAT**TAATACGACTCACTATAG**<u>GCAGCCTGGTACGCGCG</u>GTTT
AAGAGCTATGCTGGAAACA

sgMYC FWD130 GAAAT**TAATACGACTCACTATAG**<u>ATACTCACAGGACAAGGATG</u>G
TTTAAGAGCTATGCTGGAAACA

sgMYC FWD131 GAAAT**TAATACGACTCACTATAG**<u>GGAGCAGCAGAGAAAGGGAG</u>
GTTTAAGAGCTATGCTGGAAACA

sgMYC FWD132 GAAAT**TAATACGACTCACTATAG**<u>GCGCGCGTAGTTAATTCATG</u>GT
TTAAGAGCTATGCTGGAAACA

sgMYC FWD133 GAAAT**TAATACGACTCACTATAG**<u>GGTGGGGAGGAGACTCAGCC</u>
GTTTAAGAGCTATGCTGGAAACA

sgMYC FWD134 GAAAT**TAATACGACTCACTATAG**<u>GGGTTCCCAAAGCAGA</u>GTTTAA
GAGCTATGCTGGAAACA

sgMYC FWD135 GAAAT**TAATACGACTCACTATAG**G<u>TTATAATGCGAGGGTCTGGA</u>
GTTTAAGAGCTATGCTGGAAACA

sgMYC FWD136	GAAAT**TAATACGACTCACTATAG**GAGAAGGGCAGGGCTTCTCAGGTTTAAGAGCTATGCTGGAAACA
sgMYC FWD137	GAAAT**TAATACGACTCACTATAG**GGGAAAAAGAACGGAGGGAGTTTAAGAGCTATGCTGGAAACA
sgMYC FWD138	GAAAT**TAATACGACTCACTATAG**GCTGTAGTAATTCCAGCGAGGTTTAAGAGCTATGCTGGAAACA
sgMYC FWD139	GAAAT**TAATACGACTCACTATAG**GGGCGAGCAGAGCTGCGCTGGTTTAAGAGCTATGCTGGAAACA
sgMYC FWD140	GAAAT**TAATACGACTCACTATAG**GGAGATCCGGAGCGAATAGGGTTTAAGAGCTATGCTGGAAACA
sgMYC FWD141	GAAAT**TAATACGACTCACTATAG**ACCGCTGGCTGGGGGATCAGGTTTAAGAGCTATGCTGGAAACA
sgMYC FWD142	GAAAT**TAATACGACTCACTATAG**GAAACTTTGCCCATAGCAGGTTTAAGAGCTATGCTGGAAACA
sgMYC FWD143	GAAAT**TAATACGACTCACTATAG**GACGCGACTCTCCCGACGCGGTTTAAGAGCTATGCTGGAAACA
sgMYC FWD144	GAAAT**TAATACGACTCACTATAG**GCGGGTCCTGGCAGCGGCGGTTTAAGAGCTATGCTGGAAACA
sgMYC FWD145	GAAAT**TAATACGACTCACTATAG**GCAGCTGCTTAGACGCGTTTAAGAGCTATGCTGGAAACA
sgMYC FWD146	GAAAT**TAATACGACTCACTATAG**GATGAGTCGAATGCCTAAATAGTTTAAGAGCTATGCTGGAAACA
sgMYC FWD147	GAAAT**TAATACGACTCACTATAG**GAGAAAAGTGTCAATAGCGCGTTTAAGAGCTATGCTGGAAACA
sgMYC FWD148	GAAAT**TAATACGACTCACTATAG**GGTAATCCAGAACTGGATCGGTTTAAGAGCTATGCTGGAAACA
sgMYC FWD149	GAAAT**TAATACGACTCACTATAG**GATGGGAGAGGAGAAGGCAGGTTTAAGAGCTATGCTGGAAACA
sgMYC FWD150	GAAAT**TAATACGACTCACTATAG**ATAAGGCAGAAATCTCGAAAGTTTAAGAGCTATGCTGGAAACA
sgMYC FWD151	GAAAT**TAATACGACTCACTATAG**ATAAAGCAGGAATGTCCGACG

TTTAAGAGCTATGCTGGAAACA

sgMYC FWD152 GAAAT**TAATACGACTCACTATAG**GCTGGGGGTTGCTTTGCGGTG
TTTAAGAGCTATGCTGGAAACA

sgMYC FWD153 GAAAT**TAATACGACTCACTATAG**GGCTCACACAGGCGATATGGT
TTAAGAGCTATGCTGGAAACA

sgMYC FWD154 GAAAT**TAATACGACTCACTATAG**GACTTGTCCCCGTCTCCGGGG
TTTAAGAGCTATGCTGGAAACA

sgMYC FWD155 GAAAT**TAATACGACTCACTATAG**ACAGCCGGAGACGGACACTGG
TTTAAGAGCTATGCTGGAAACA

sgMYC FWD156 GAAAT**TAATACGACTCACTATAG**GGCGGGTTGGAATCGCCGCG
GTTTAAGAGCTATGCTGGAAACA

sgMYC FWD157 GAAAT**TAATACGACTCACTATAG**ATTTAAAACCTGGGTCTCTAGG
TTTAAGAGCTATGCTGGAAACA

sgMYC FWD158 GAAAT**TAATACGACTCACTATAG**GTGTTGGGTAGGCGCAGGCAG
GTTTAAGAGCTATGCTGGAAACA

sgMYC FWD159 GAAAT**TAATACGACTCACTATAG**GTCGTTGACTTGGAAAAACCAG
TTTAAGAGCTATGCTGGAAACA

sgMYC FWD160 GAAAT**TAATACGACTCACTATAG**AGCCCTGACTCCCCTGCCGGT
TTAAGAGCTATGCTGGAAACA

sgMYC FWD161 GAAAT**TAATACGACTCACTATAG**GAGATGCGGAGGAACTGCGGT
TTAAGAGCTATGCTGGAAACA

sgMYC FWD162 GAAAT**TAATACGACTCACTATAG**GCCCCGGAGCCACCCCACCAA
GTTTAAGAGCTATGCTGGAAACA

sgMYC FWD163 GAAAT**TAATACGACTCACTATAG**GCATCTCCGTATTGAGTGCGAA
GTTTAAGAGCTATGCTGGAAACA

sgMYC FWD164 GAAAT**TAATACGACTCACTATAG**GGAGGGGTGTTAAAGCCCGGT
TTAAGAGCTATGCTGGAAACA

sgMYC FWD165 GAAAT**TAATACGACTCACTATAG**GGAGAAGGCGAGAGGCGCCT
GTTTAAGAGCTATGCTGGAAACA

sgMYC FWD166 GAAAT**TAATACGACTCACTATAG**GAAAACAATTTGCCAAAATCCA
GTTTAAGAGCTATGCTGGAAACA

sgMYC FWD167	GAAAT**TAATACGACTCACTATAG**GCGGCTTCTTAAGGGCGCCAGTTTAAGAGCTATGCTGGAAACA
sgMYC FWD168	GAAAT**TAATACGACTCACTATAG**GCGCTCCGGGCTCCCGGGTTTAAGAGCTATGCTGGAAACA
sgMYC FWD169	GAAAT**TAATACGACTCACTATAG**GTGCGTCTCCGAGATAGCAGGTTTAAGAGCTATGCTGGAAACA
sgMYC FWD170	GAAAT**TAATACGACTCACTATAG**GGTCTTGGTGGGGGAATAAAGTTTAAGAGCTATGCTGGAAACA
sgMYC FWD171	GAAAT**TAATACGACTCACTATAG**GGGGAGAGGTTCGGGACTGGTTTAAGAGCTATGCTGGAAACA
sgMYC FWD172	GAAAT**TAATACGACTCACTATAG**GAGGCAGTCTTGAGTTAAAGGTTTAAGAGCTATGCTGGAAACA
sgMYC FWD173	GAAAT**TAATACGACTCACTATAG**GTTGGTGAAGCTAACGTTGAGTTTAAGAGCTATGCTGGAAACA
sgMYC FWD174	GAAAT**TAATACGACTCACTATAG**GTATTTCTACTGCGACGAGGGTTTAAGAGCTATGCTGGAAACA
sgMYC FWD175	GAAAT**TAATACGACTCACTATAG**GATATCCTCGCTGGGCGCCGGTTTAAGAGCTATGCTGGAAACA
sgMYC FWD176	GAAAT**TAATACGACTCACTATAG**GCGAGCAGAGCCCGGAGCGGGTTTAAGAGCTATGCTGGAAACA
sgMYC FWD177	GAAAT**TAATACGACTCACTATAG**GCTTCGGGGAGACAACGACGGGTTTAAGAGCTATGCTGGAAACA
sgMYC FWD178	GAAAT**TAATACGACTCACTATAG**GCTCGGTCACCATCTCCAGCGTTTAAGAGCTATGCTGGAAACA
sgMYC FWD179	GAAAT**TAATACGACTCACTATAG**ATCATCATCCAGGACTGTATGGTTTAAGAGCTATGCTGGAAACA
sgMYC FWD180	GAAAT**TAATACGACTCACTATAG**GCTCTGAGACGAGCTTGGCGGGTTTAAGAGCTATGCTGGAAACA
sgMYC FWD181	GAAAT**TAATACGACTCACTATAG**GGCTGCGCGCAAAGACAGGTTTAAGAGCTATGCTGGAAACA
sgMYC FWD182	GAAAT**TAATACGACTCACTATAG**GGAGCAGACGCTGTGGCCGGT

	TTAAGAGCTATGCTGGAAACA
sgMYC FWD183	GAAAT**TAATACGACTCACTATAG**GGTAGGGGAAGACCACCGAGGTTTAAGAGCTATGCTGGAAACA
sgMYC FWD184	GAAAT**TAATACGACTCACTATAG**GCTGGAGTCTTGCGAGGCGCGTTTAAGAGCTATGCTGGAAACA
sgMYC FWD185	GAAAT**TAATACGACTCACTATAG**GAGGAGAGCAGAGAATCCGGTTTAAGAGCTATGCTGGAAACA
sgMYC FWD186	GAAAT**TAATACGACTCACTATAG**GTCTCCTCATGGAGCACCAGTTTAAGAGCTATGCTGGAAACA
sgMYC FWD187	GAAAT**TAATACGACTCACTATAG**GGCCTGTCAAAAGTGGGGTTTAAGAGCTATGCTGGAAACA
sgMYC FWD188	GAAAT**TAATACGACTCACTATAG**AATAAGCTGCCAATGAAAATGTTTAAGAGCTATGCTGGAAACA
sgMYC FWD189	GAAAT**TAATACGACTCACTATAG**GCTAAAGCCCAAGGTTTCAGGTTTAAGAGCTATGCTGGAAACA
sgMYC FWD190	GAAAT**TAATACGACTCACTATAG**GCAAACATGGGCAGTCTAAGGTTTAAGAGCTATGCTGGAAACA
sgMYC FWD191	GAAAT**TAATACGACTCACTATAG**GAGTTGTAAGATAAGCCAGAGTTTAAGAGCTATGCTGGAAACA
sgMYC FWD192	GAAAT**TAATACGACTCACTATAG**GCAATTAAAATGTTAACGGGGTTTAAGAGCTATGCTGGAAACA
sgMYC FWD193	GAAAT**TAATACGACTCACTATAG**GTATGAATGAGGATAAGAGGTTTAAGAGCTATGCTGGAAACA
sgMYC FWD194	GAAAT**TAATACGACTCACTATAG**GCCACTTCTCGGAAGTTAAGAGTTTAAGAGCTATGCTGGAAACA
sgMYC FWD195	GAAAT**TAATACGACTCACTATAG**GTGTTTAGAGGCTAGGCAGTTTAAGAGCTATGCTGGAAACA
sgMYC FWD196	GAAAT**TAATACGACTCACTATAG**GAACTGCCTCAAGAGTGGGTGTTTAAGAGCTATGCTGGAAACA
sgMYC FWD197	GAAAT**TAATACGACTCACTATAG**GCCAAAAGTCCAAGAGGGCGGGTTTAAGAGCTATGCTGGAAACA

sgMYC FWD198 GAAAT**TAATACGACTCACTATAG**AATGATAGCTGCAAATTGCTGT
TTAAGAGCTATGCTGGAAACA

sgMYC FWD199 GAAAT**TAATACGACTCACTATAG**GTAAAGTCCCTCAAAAATAGGG
TTTAAGAGCTATGCTGGAAACA

sgMYC FWD200 GAAAT**TAATACGACTCACTATAG**GTCCAAAGCCTCATTAAGTCTT
GTTTAAGAGCTATGCTGGAAACA

sgMYC FWD201 GAAAT**TAATACGACTCACTATAG**GACAGCTGGGTTATGGCAGTT
TAAGAGCTATGCTGGAAACA

sgMYC FWD202 GAAAT**TAATACGACTCACTATAG**GCTTCATGGTGAGAGGAGTAA
GTTTAAGAGCTATGCTGGAAACA

sgMYC FWD203 GAAAT**TAATACGACTCACTATAG**GTATTTGTACAGCATTAATCGT
TTAAGAGCTATGCTGGAAACA

sgMYC FWD204 GAAAT**TAATACGACTCACTATAG**GAAATCACTCCTTTAGCAGTTT
AAGAGCTATGCTGGAAACA

sgMYC FWD205 GAAAT**TAATACGACTCACTATAG**GAGGAGGAACAAGAAGATGGT
TTAAGAGCTATGCTGGAAACA

sgMYC FWD206 GAAAT**TAATACGACTCACTATAG**GTTTCTGTGGAAAAGAGGCGTT
TAAGAGCTATGCTGGAAACA

sgMYC FWD207 GAAAT**TAATACGACTCACTATAG**GGCACCTCTTGAGGACCAGTG
TTTAAGAGCTATGCTGGAAACA

sgMYC FWD208 GAAAT**TAATACGACTCACTATAG**GCAGGATAGTCCTTCCGAGGT
TTAAGAGCTATGCTGGAAACA

sgMYC FWD209 GAAAT**TAATACGACTCACTATAG**GGTTGTTGCTGATCTGTCTCGT
TTAAGAGCTATGCTGGAAACA

sgMYC FWD210 GAAAT**TAATACGACTCACTATAG**GCCTCTTGACATTCTCCTGTTT
AAGAGCTATGCTGGAAACA

sgMYC FWD211 GAAAT**TAATACGACTCACTATAG**GAGGAGGAACGAGCTAAAAGT
TTAAGAGCTATGCTGGAAACA

sgMYC FWD212 GAAAT**TAATACGACTCACTATAG**GGAGTTGGAAAACAATGAAAGT
TTAAGAGCTATGCTGGAAACA

sgMYC FWD213 GAAAT**TAATACGACTCACTATAG**ACATCCTGTCCGTCCAAGCAG

GTTTAAGAGCTATGCTGGAAACA

sgMYC FWD214 GAAAT**TAATACGACTCACTATAG**GTGCGTAAGGAAAAGTAGTTTAAGAGCTATGCTGGAAACA

sgMYC FWD215 GAAAT**TAATACGACTCACTATAG**GCATTTGAAACAAGTTCATGTTTAAGAGCTATGCTGGAAACA

sgMYC FWD216 GAAAT**TAATACGACTCACTATAG**GTCTCAAGACTCAGCCAGTTTAAGAGCTATGCTGGAAACA

sgMYC FWD217 GAAAT**TAATACGACTCACTATAG**ACATTCACAACTTAAGATTGTTTAAGAGCTATGCTGGAAACA

sgMYC FWD218 GAAAT**TAATACGACTCACTATAG**GCAATTGATGAAAACAAACAGTTTAAGAGCTATGCTGGAAACA

sgMYC FWD219 GAAAT**TAATACGACTCACTATAG**GAGTTTTCCTCTGTTGAAATGTTTAAGAGCTATGCTGGAAACA

sgMYC FWD220 GAAAT**TAATACGACTCACTATAG**GAGGTTCTAAGATGCTTCCGTTTAAGAGCTATGCTGGAAACA

sgMYC FWD221 GAAAT**TAATACGACTCACTATAG**GTAGGCAAAGGAGATACAAGGTTTAAGAGCTATGCTGGAAACA

sgMYC FWD222 GAAAT**TAATACGACTCACTATAG**GGGAGTTGGGAGGAAGGTGGTTTAAGAGCTATGCTGGAAACA

sgMYC FWD223 GAAAT**TAATACGACTCACTATAG**ATTCCTGGGTTTGGAGTGAGCAGTTTAAGAGCTATGCTGGAAACA

sgMYC FWD224 GAAAT**TAATACGACTCACTATAG**ACCAAGGCATGATAGCGAAGTTTAAGAGCTATGCTGGAAACA

sgMYC FWD225 GAAAT**TAATACGACTCACTATAG**AGGTTTAGGACCTAAGTTTAAGAGCTATGCTGGAAACA

sgMYC FWD226 GAAAT**TAATACGACTCACTATAG**GTTTCCCCTTGACCATGAGGAGTTTAAGAGCTATGCTGGAAACA

sgMYC FWD227 GAAAT**TAATACGACTCACTATAG**GCTGGCTGCTTGTGAGTACGTTTAAGAGCTATGCTGGAAACA

sgMYC FWD228 GAAAT**TAATACGACTCACTATAG**AACATCTAAGCCTGGTCGTTTAAGAGCTATGCTGGAAACA

sgMYC FWD229	GAAAT**TAATACGACTCACTATAG**GGCTAAGGTAGGAGTCAAGAGTTTAAGAGCTATGCTGGAAACA
sgMYC FWD230	GAAAT**TAATACGACTCACTATAG**ATCTGAACTGGCTTCTTCCCGTTTAAGAGCTATGCTGGAAACA
sgMYC FWD231	GAAAT**TAATACGACTCACTATAG**GTAAAAAAGGATGGAAGCAGTTTAAGAGCTATGCTGGAAACA
sgMYC FWD232	GAAAT**TAATACGACTCACTATAG**GTTCAAAAATACCTTTTCAGTTTAAGAGCTATGCTGGAAACA
sgMYC FWD233	GAAAT**TAATACGACTCACTATAG**ATTTCTGGTTTGGGCCATGTTTAAGAGCTATGCTGGAAACA
sgMYC FWD234	GAAAT**TAATACGACTCACTATAG**ATTGTCTCAGTCTCAAAGTGTTTAAGAGCTATGCTGGAAACA
sgMYC FWD235	GAAAT**TAATACGACTCACTATAG**ATTTATTGATTTATGGGTGGGTTTAAGAGCTATGCTGGAAACA
sgMYC FWD236	GAAAT**TAATACGACTCACTATAG**GGATCAAGAAAAAGACATTAGTTTAAGAGCTATGCTGGAAACA
sgMYC FWD237	GAAAT**TAATACGACTCACTATAG**GTCATATAGGCGAATTTCAAAGTTTAAGAGCTATGCTGGAAACA
sgMYC FWD238	GAAAT**TAATACGACTCACTATAG**GCTCAGTCTTTGCCCCTTTGGTTTAAGAGCTATGCTGGAAACA
sgMYC FWD239	GAAAT**TAATACGACTCACTATAG**GACTCACTTGGGAATCGGGAGTTTAAGAGCTATGCTGGAAACA
sgMYC FWD240	GAAAT**TAATACGACTCACTATAG**GAACACTCTCTCCTATTCTGGTTTAAGAGCTATGCTGGAAACA
sgMYC FWD241	GAAAT**TAATACGACTCACTATAG**GGAAAGAACTTTAGGGATGGTTTAAGAGCTATGCTGGAAACA
sgMYC FWD242	GAAAT**TAATACGACTCACTATAG**GGTAAGAGCGGCCTAATGTTTAAGAGCTATGCTGGAAACA
sgMYC FWD243	GAAAT**TAATACGACTCACTATAG**GCCTGACTTTCGGGAAGGAAGTGTTTAAGAGCTATGCTGGAAACA
sgMYC FWD244	GAAAT**TAATACGACTCACTATAG**GCCTATACAGGGAGTCCCAGG

TTTAAGAGCTATGCTGGAAACA

sgMYC FWD245 GAAAT**TAATACGACTCACTATAG**ATCAAGAATCGGACGTGAAGTT
TAAGAGCTATGCTGGAAACA

sgMYC FWD246 GAAAT**TAATACGACTCACTATAG**GGATAGTGTCATGGATAAAGTT
TAAGAGCTATGCTGGAAACA

sgMYC FWD247 GAAAT**TAATACGACTCACTATAG**GGAATGATTTTGTTGAGGGAGT
TTAAGAGCTATGCTGGAAACA

sgMYC FWD248 GAAAT**TAATACGACTCACTATAG**GATCTCCTTTGTTGCTTCCAAA
GTTTAAGAGCTATGCTGGAAACA

sgMYC FWD249 GAAAT**TAATACGACTCACTATAG**GCTCTCTAAGTATTAGGCTGTT
TAAGAGCTATGCTGGAAACA

sgMYC FWD250 GAAAT**TAATACGACTCACTATAG**GACAAACTCTCACACAAAACTG
TTTAAGAGCTATGCTGGAAACA

sgMYC FWD251 GAAAT**TAATACGACTCACTATAG**GTTTGCAATAACTATAATGTTTA
AGAGCTATGCTGGAAACA

sgMYC FWD252 GAAAT**TAATACGACTCACTATAG**GATGGGAAAAAATGCTACAGG
TTTAAGAGCTATGCTGGAAACA

sgMYC FWD253 GAAAT**TAATACGACTCACTATAG**GTGCATTTATAGACAAGGGGTT
TAAGAGCTATGCTGGAAACA

sgMYC FWD254 GAAAT**TAATACGACTCACTATAG**GAATATTATAAGACTACATTAA
GTTTAAGAGCTATGCTGGAAACA

sgMYC FWD255 GAAAT**TAATACGACTCACTATAG**GCCAGTAGGATGGGAGCAGTT
TAAGAGCTATGCTGGAAACA

sgMYC FWD256 GAAAT**TAATACGACTCACTATAG**GACTTTTTGCTAAGGCTTTGGG
TTTAAGAGCTATGCTGGAAACA

sgMYC FWD257 GAAAT**TAATACGACTCACTATAG**GTTTTCCTCTAAATGGAGAGTG
TTTAAGAGCTATGCTGGAAACA

sgMYC FWD258 GAAAT**TAATACGACTCACTATAG**GCTTTTTCGGAAGACAGAGTTG
AGTTTAAGAGCTATGCTGGAAACA

sgMYC FWD259 GAAAT**TAATACGACTCACTATAG**GGGACTGTGGCTGAGGTCCCG
TTTAAGAGCTATGCTGGAAACA

sgMYC FWD260 GAAAT**TAATACGACTCACTATAG**ATAGACCTCAGATTGCACGTTTAAGAGCTATGCTGGAAACA

sgMYC FWD261 GAAAT**TAATACGACTCACTATAG**GAGTGTATGTATGTAATAAGTTTAAGAGCTATGCTGGAAACA

sgMYC FWD262 GAAAT**TAATACGACTCACTATAG**GGAGGAGGTTCAAGCTCCGTTTAAGAGCTATGCTGGAAACA

sgMYC FWD263 GAAAT**TAATACGACTCACTATAG**GAATCCAGGAAAGAGCCCGTTTAAGAGCTATGCTGGAAACA

sgMYC FWD264 GAAAT**TAATACGACTCACTATAG**GAGAGATAAGGAGAAGCTGTTTAAGAGCTATGCTGGAAACA

sgMYC FWD265 GAAAT**TAATACGACTCACTATAG**GGAAGCCCTGGTGTGTCAAGTTTAAGAGCTATGCTGGAAACA

sgMYC FWD266 GAAAT**TAATACGACTCACTATAG**ATCACATGAACGCAGCTTCCGTTTAAGAGCTATGCTGGAAACA

sgMYC FWD267 GAAAT**TAATACGACTCACTATAG**GAATGAAGGAGAGGATGCCGGGTTTAAGAGCTATGCTGGAAACA

sgMYC FWD268 GAAAT**TAATACGACTCACTATAG**GAGAAGGTAGGGCAGGGTGCTGTTTAAGAGCTATGCTGGAAACA

sgMYC FWD269 GAAAT**TAATACGACTCACTATAG**GAAGAGGATCACTGGGAATGAGTTTAAGAGCTATGCTGGAAACA

sgMYC FWD270 GAAAT**TAATACGACTCACTATAG**GGAGGCAGCATAGGACTGAGGTTTAAGAGCTATGCTGGAAACA

sgMYC FWD271 GAAAT**TAATACGACTCACTATAG**AATGAAGTAGGTAGACAAAAGTTTAAGAGCTATGCTGGAAACA

sgMYC FWD272 GAAAT**TAATACGACTCACTATAG**GCAGGAATGATTCAATCTAGGTTTAAGAGCTATGCTGGAAACA

sgMYC FWD273 GAAAT**TAATACGACTCACTATAG**GTGATGGTGCTACCAACCGAAGTTTAAGAGCTATGCTGGAAACA

sgMYC FWD274 GAAAT**TAATACGACTCACTATAG**GCTACATTCTATGTAGCTCTCGTTTAAGAGCTATGCTGGAAACA

sgMYC FWD275 GAAAT**TAATACGACTCACTATAG**AGGATGACGGAGGAGAAAGAG

	TTTAAGAGCTATGCTGGAAACA
sgMYC FWD276	GAAAT**TAATACGACTCACTATAG**ATTGTGATCAAGATAACCAGTTTAAGAGCTATGCTGGAAACA
sgMYC FWD277	GAAAT**TAATACGACTCACTATAG**AATAGCCTGGTGAATGAGAAGTTTAAGAGCTATGCTGGAAACA
sgMYC FWD278	GAAAT**TAATACGACTCACTATAG**AATTCTAATGGGGAAGCAGGGTTTAAGAGCTATGCTGGAAACA
sgMYC FWD279	GAAAT**TAATACGACTCACTATAG**GCTAGAACAGACCCAATTACAGTTTAAGAGCTATGCTGGAAACA
sgMYC FWD280	GAAAT**TAATACGACTCACTATAG**GTACCTGCCTGGTTGCTTGAGTTTAAGAGCTATGCTGGAAACA
sgMYC FWD281	GAAAT**TAATACGACTCACTATAG**GGATGATGACTCAGAGCGTTTAAGAGCTATGCTGGAAACA
sgMYC FWD282	GAAAT**TAATACGACTCACTATAG**GGAAGATTGCGTCTTCTCCAGTTTAAGAGCTATGCTGGAAACA
sgMYC FWD283	GAAAT**TAATACGACTCACTATAG**AATCAGTTAGGATGCAACCAGTTTAAGAGCTATGCTGGAAACA
sgMYC FWD284	GAAAT**TAATACGACTCACTATAG**GCATCTCAGAAGACCTGGTTTAAGAGCTATGCTGGAAACA
sgMYC FWD285	GAAAT**TAATACGACTCACTATAG**GTCTCCCCCTCTCCCATAGCAGTTTAAGAGCTATGCTGGAAACA
sgMYC FWD286	GAAAT**TAATACGACTCACTATAG**GCCCACCCATGGGTCTCCCCCAGTTTAAGAGCTATGCTGGAAACA
sgMYC FWD287	GAAAT**TAATACGACTCACTATAG**GCAGTCCAAACTGTTTCACCAGTTTAAGAGCTATGCTGGAAACA
sgMYC FWD288	GAAAT**TAATACGACTCACTATAG**GGTCACATTGATCCCACCAGTTTAAGAGCTATGCTGGAAACA

Bold, T7 promoter；underlined, sequence of sgRNA target; FWD, forward primer.
