# Supplementary material for: Simple and versatile imaging of genomic loci in live mammalian cells and early pre-implantation embryos using CAS-LiveFISH
Source: Sci Rep. 2021 Jun 9;11:12220. doi: 10.1038/s41598-021-91787-y (PMC8190065; doi:10.1038/s41598-021-91787-y)
Supplement: Supplementary file 2 — Supplementary Video Legend. [file 41598_2021_91787_MOESM2_ESM.docx]

**SUPPLEMENTARY VIDEO LEGENDS**

**Supplementary Video 1. Real-time tracking of HPV-Alexa 647 and TMR-MUC4 loci in a live HeLa cell.** Top panels: whole nucleus views; raw images after background subtraction with a rolling-ball filter. Bottom panels: ROIs indicated by yellow arrows; processed images after band-pass filtering of the raw images. Video is played at 2-fold faster speed than the acquired data.
